# Supplementary material for: Strategies for robust, accurate, and generalizable benchmarking of drug discovery platforms
Source: Bioinformatics. 2025 Nov 5;41(11):btaf604. doi: 10.1093/bioinformatics/btaf604 (PMC12607264; doi:10.1093/bioinformatics/btaf604)
Supplement: btaf604_Supplementary_Data [file btaf604_supplementary_data.zip › suppF_correl.pdf]

Supplementary figure 1 – Indication size

CTD

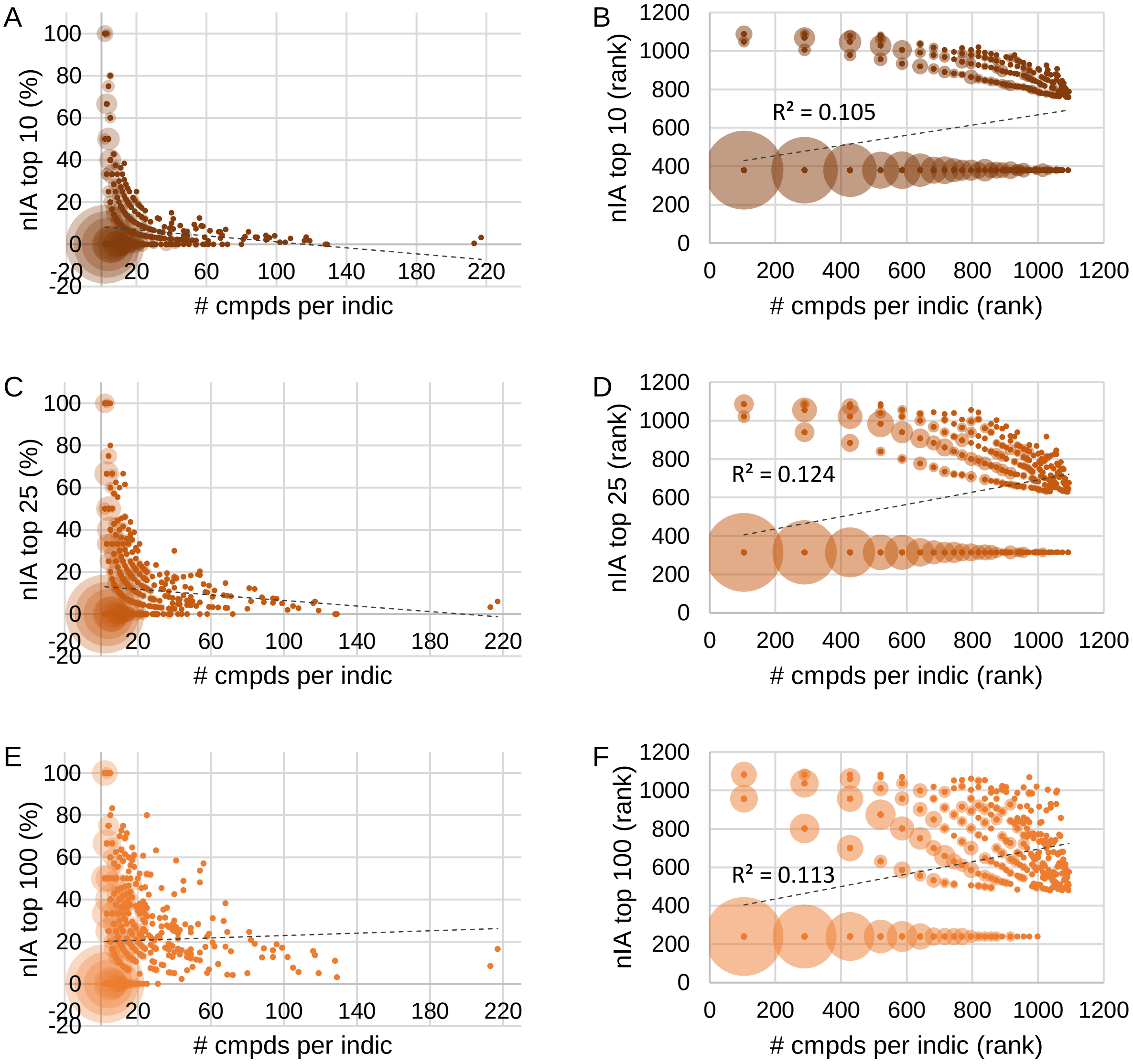

TTD

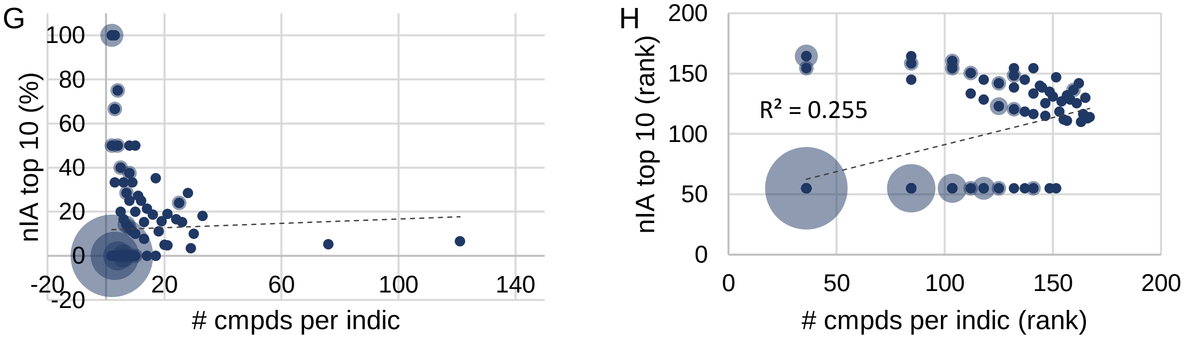

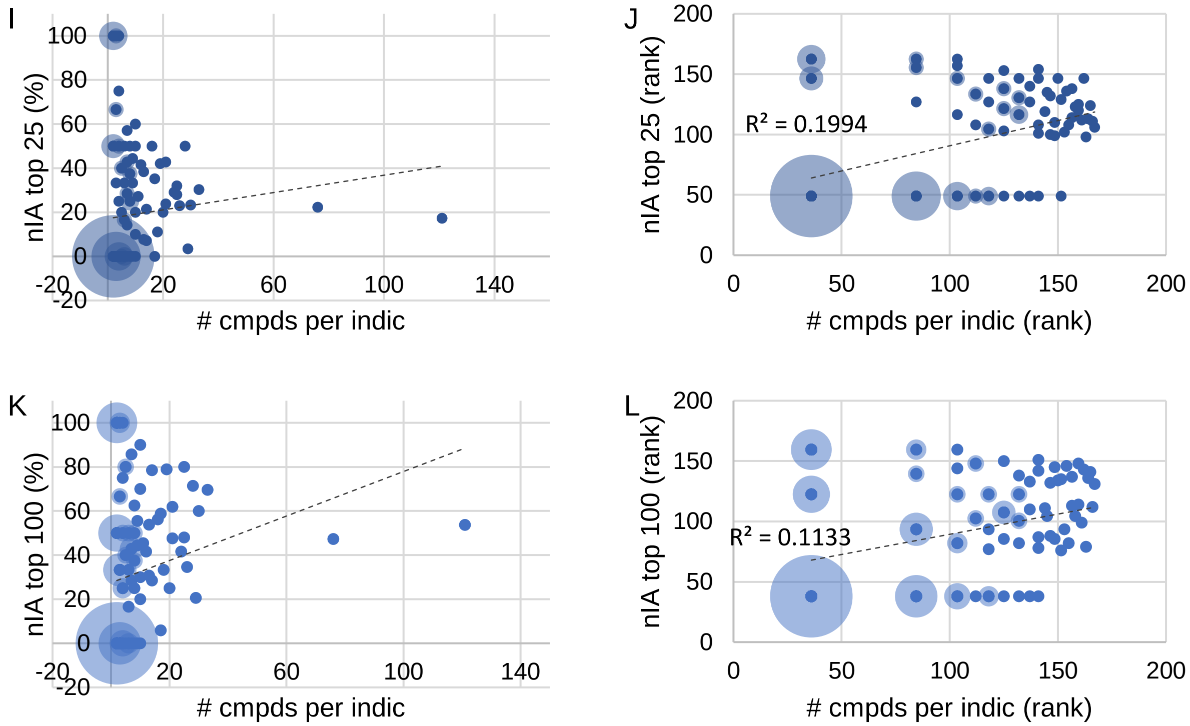

I
nIA top 25 (%)
cmpds per indic
J
nIA top 25 (rank)
R² = 0.1994
# cmpds per indic (rank)
K
nIA top 100 (%)
cmpds per indic
L
nIA top 100 (rank)
R² = 0.1133
# cmpds per indic (rank)

Supplementary figure 2 – nIA versus IA

CTD

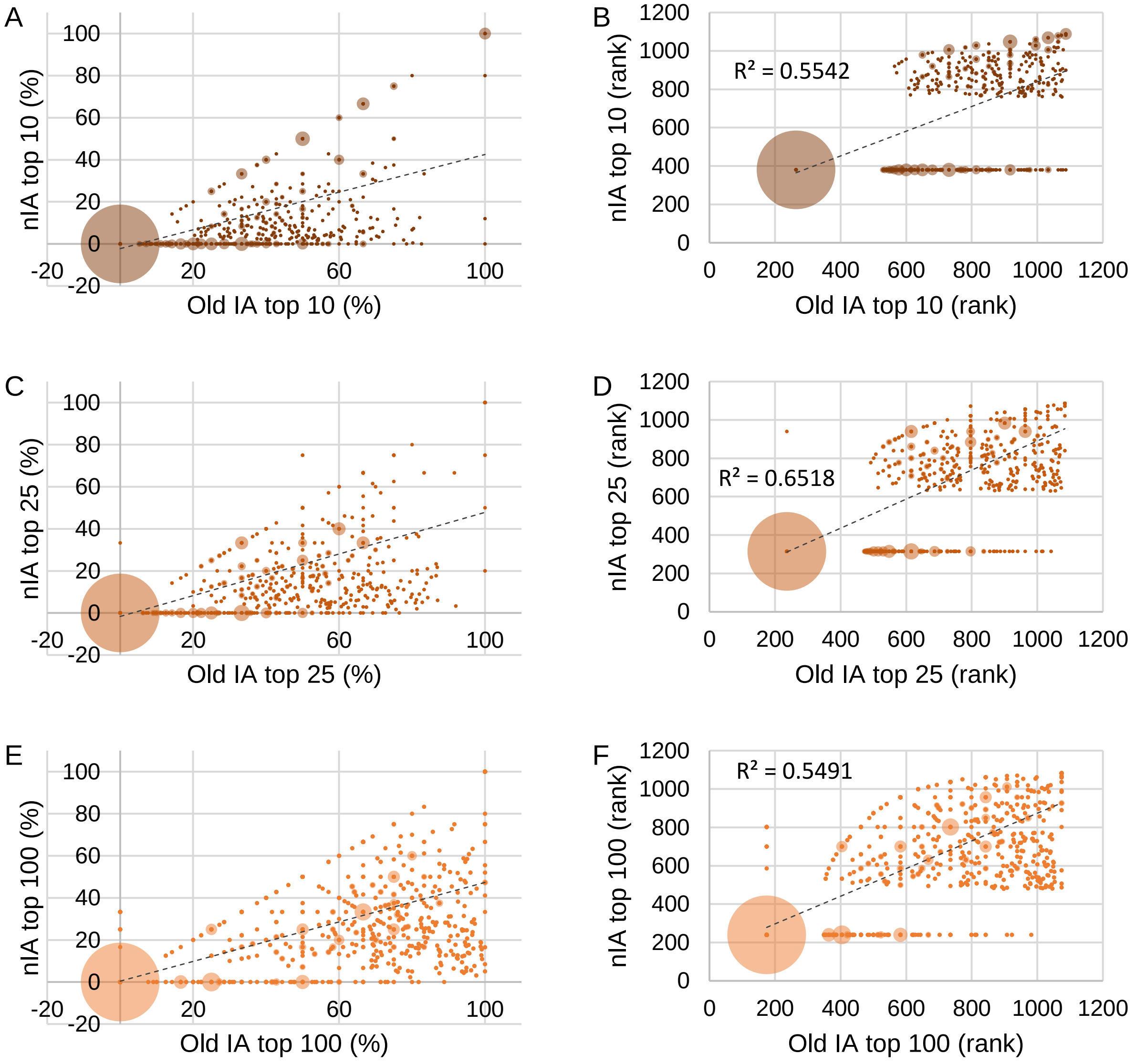

TTD

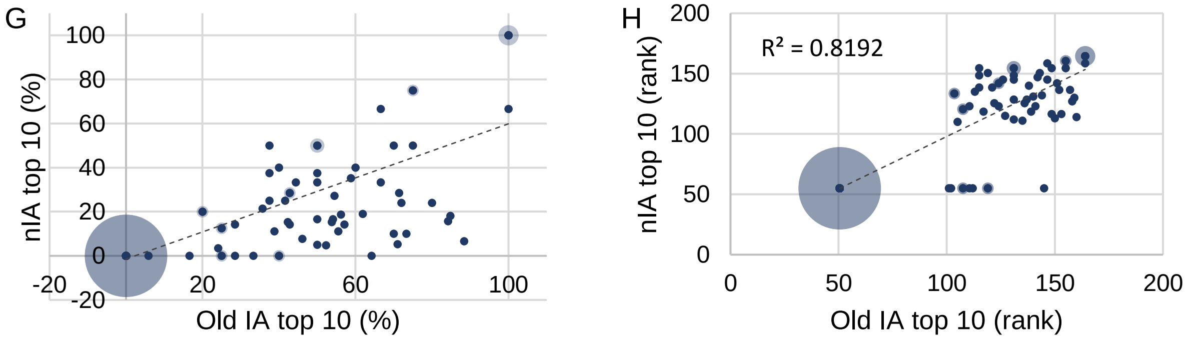

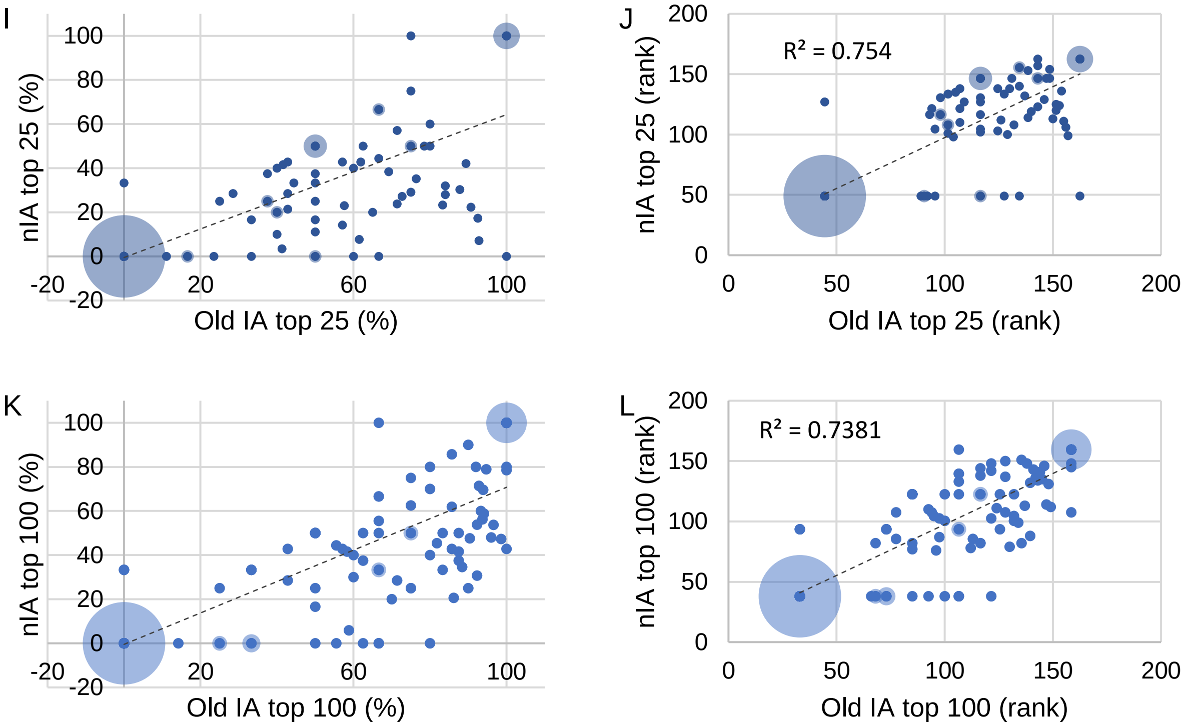
I
nIA top 25 (%)
Old IA top 25 (%)
J
$R^2 = 0.754$
nIA top 25 (rank)
Old IA top 25 (rank)
K
nIA top 100 (%)
Old IA top 100 (%)
L
$R^2 = 0.7381$
nIA top 100 (rank)
Old IA top 100 (rank)

Supplementary figure 3 – Compound similarity

CTD

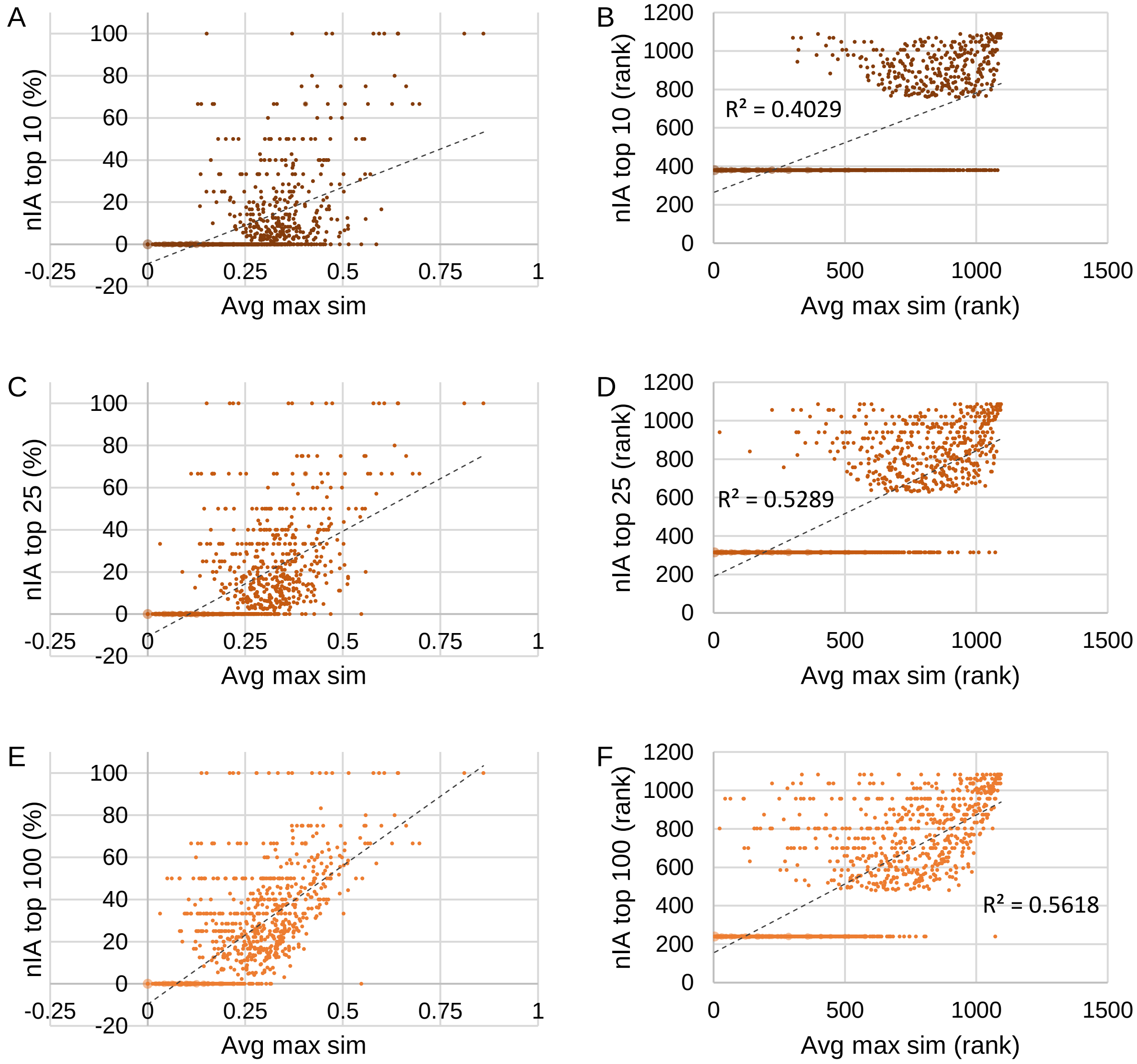

TTD

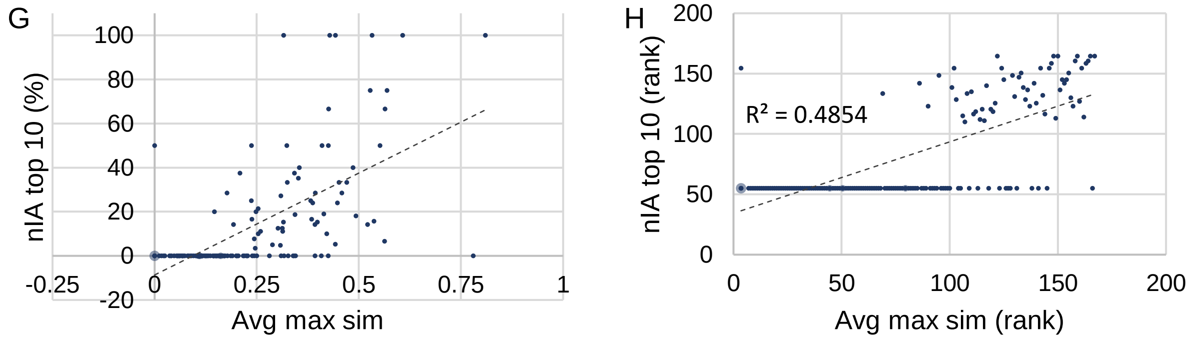

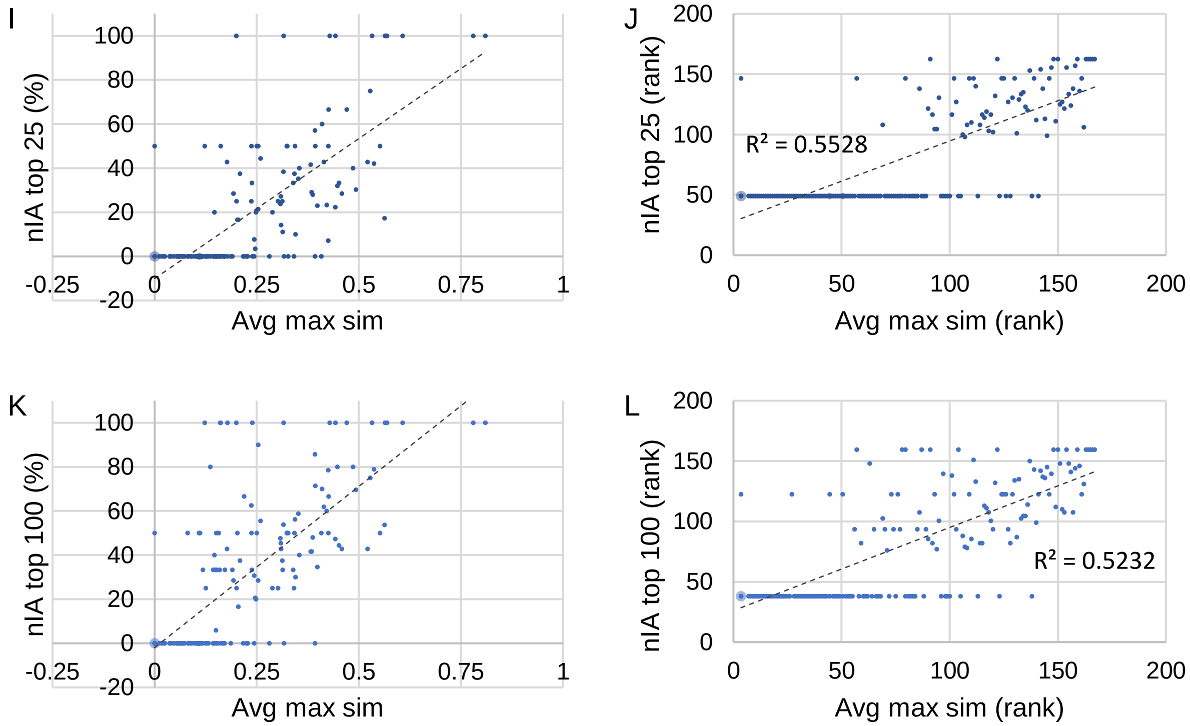
I
nIA top 25 (%)
Avg max sim
J
nIA top 25 (rank)
Avg max sim (rank)
R² = 0.5528
K
nIA top 100 (%)
Avg max sim
L
nIA top 100 (rank)
Avg max sim (rank)
R² = 0.5232
